# Ubiquitination as a Key Regulator of Endosomal Signaling by GPCRs

**DOI:** 10.3389/fcell.2019.00043

**Published:** 2019-03-29

**Authors:** Jeremy C. Burton, Neil J. Grimsey

**Affiliations:** Department of Pharmaceutical and Biomedical Sciences, College of Pharmacy, University of Georgia, Athens, GA, United States

**Keywords:** GPCR (G-protein-coupled receptors), p38 activation, E3 ligase, Ubiquitin (Ub), NEDD4 E3 ubiquitin ligases, Nedd4-2, TAB1

## Abstract

G protein-coupled receptors (GPCRs) represent the largest family of therapeutic targets for FDA approved drugs. Therefore, understanding the molecular regulation of their signaling pathways is of paramount importance. Similarly, the mitogen activated protein kinase (MAPK) p38 is a critical mediator of proinflammatory disease. Yet despite decades of intense investigation, therapeutically viable inhibitors have struggled to make it into the clinic. New studies describing the regulation and activation of a GPCR dependent atypical p38 signaling pathway represents a novel therapeutic avenue to the treatment of many proinflammatory disorders. These recent studies have defined how thrombin and ADP can induce Src dependent activation of the E3 ubiquitin ligase NEDD4-2. Src dependent phosphorylation of a 2,3-linker peptide releases NEDD4-2 auto-inhibition and triggers the induction of proinflammatory atypical p38 signaling from the endosome. Activation of the atypical p38 pathway requires the direct interaction between an adaptor protein TAB1 and p38, that bypasses the requirement for the classical MKK3/6 dependent activation of p38. Therefore, providing a mechanism to specifically block proinflammatory GPCR atypical p38 activation while leaving basic p38 activity intact. Critically, new studies demonstrated that disruption of the TAB1-p38 interface is a druggable target, that would enable the selective inhibition of proinflammatory p38 signaling and ischemic injury. Atypical p38 signaling is linked to multiple clinically relevant pathologies including inflammation, cardiotoxicity, myocardial ischemia and ischemia reperfusion injury. Therefore, GPCR induced endosomal p38 signaling represents a novel understudied branch of proinflammatory p38 signaling and an ideal potential therapeutic target that warrants further investigation.

## Introduction

There are over 800 G protein–coupled receptors (GPCRs) expressed throughout the body, playing critical roles in many physiological functions ranging from the control of taste and sight to the regulation of neuronal function and inflammatory responses. GPCRs are sensors for extracellular stimuli, and as such are activated by a wide array of ligands to induce intracellular signaling cascades, principally through the activation of the heterotrimeric G proteins. It is perhaps not surprising then that dysregulation in their expression or activities are lead causes in the onset and progression of many human diseases including hypertension, cancer and chronic inflammatory diseases such as rheumatoid arthritis and chronic obstructive pulmonary disease (Popovic et al., 2014). Consequently, GPCRs represent the largest family of proteins targeted by current FDA approved drugs (Sriram and Insel, 2018).

**Figure 1:**
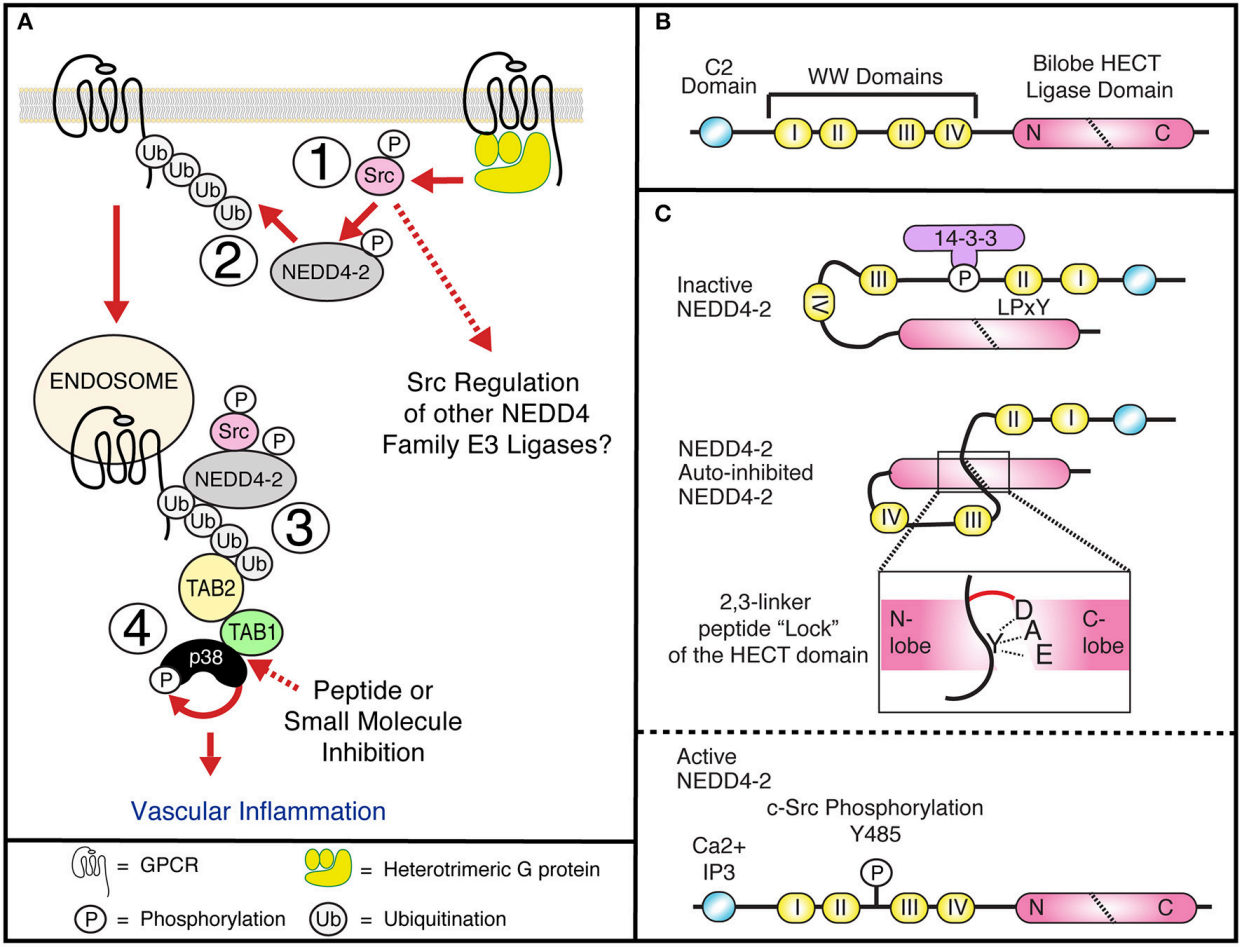
**(A1)** GPCRs induce Gα_q_ dependent c-Src activation at the plasma membrane and tyrosine phosphorylation of NEDD4-2, releasing NEDD4-2 auto-inhibition. **(A2)** Activated NEDD4-2 ubiquitinated GPCRs. **(A3)** Internalized, endosomal GPCRs recruit the adaptor proteins TAB1 and TAB2, to active p38 through an atypical pathway. **(A4)** GPCR induced atypical p38 signaling regulates proinflammatory signaling in vasculature. The interface between TAB1 and p38 is a potential druggable target for therapeutic intervention. **(B)** NEDD4-2 contains a C2 lipid and Calcium binding domain, four WW domains that bind PP/LPxY motifs and a C-terminal bilobed Catalytic HECT domain. **(C)** NEDD4-2 can be held in an inactive state by serine phosphorylation dependent recruitment of 14-3-3 (Debonneville et al., 2001; Bhalla et al., 2005; Ichimura et al., 2005), and intramolecular binding of WWII with a LPxY motif on the HECT domain (Bruce et al., 2008; Escobedo et al., 2014), or through binding of an auto-inhibitory 2,3-linker peptide that wraps the N-lobe of the HECT domain locking the ligase in a closed confirmation. Active NEDD4-2 requires dephosphorylation and release of 14-3-3, calcium and lipid binding to the C2 domain and tyrosine phosphorylation of Y485 (Grimsey et al., 2018).

It is therefore understandable that the mechanisms that control GPCR expression and signaling have been extensively studied. As with many proteins, receptor phosphorylation is an integral step in regulating GPCR activation through the induction of conformational changes and regulating adaptor protein interactions; differential phosphorylation motifs determine specific functional responses at temporally distinct steps after receptor activation i.e., the phospho-barcode (Nobles et al., 2011). Contrary to phosphorylation the role and regulation of GPCR ubiquitination has been understudied. GPCRs are 7 pass transmembrane receptors, that couple to the heterotrimeric G-proteins to induce intracellular signaling responses. Over 40 GPCRs are known to be ubiquitinated, regulating receptor expression, trafficking, and degradation through the recruitment of adaptor proteins (Jean-Charles et al., 2016). Additionally, new studies have shown a clear link for receptor ubiquitination and the activation of endosomal signaling, as discussed below (Figure 1A) (Grimsey et al., 2015, 2018).

Therefore, defining the molecular mechanisms for the activation and regulation of GPCR ubiquitination and the adaptor proteins that control GPCR ubiquitin-driven events is likely to reveal novel therapeutic targets for the regulation of human disease.

### Ubiquitination

Ubiquitin is an evolutionarily conserved small 7 kilodalton protein expressed in all biological systems. In turn, physiological and cellular functions in all species are intrinsically tied to ubiquitin-dependent regulatory pathways, and many human diseases are linked to aberrant processing of ubiquitin driven signaling or protein expression (Popovic et al., 2014). At a cellular and protein level, the covalent binding of ubiquitin either as a single moiety or as poly-ubiquitin chains causes a change in protein fate or function. Classically, the principal function of protein ubiquitination is to act as a targeting signal to induce the degradation of proteins, in part through the regulation of protein-protein interactions. However, ubiquitination can orchestrate a large array of responses through the specific type of ubiquitin modification. Signal ubiquitin moieties can be added to proteins, termed mono/multi-mono ubiquitination to regulate protein-protein interactions and localization (Rotin and Kumar, 2009). Alternatively, ubiquitin contains 7 lysine residues (K6, K11, K27, K29, K33, K48, and K63) once bound to a substrate, additional ubiquitin moieties can then be conjugated onto any one of these lysine residues. These form polyubiquitin chains with each subsequent moiety conjugated at the same residue i.e., K63-K63 or K48-K48 polyubiquitin chains, or they can form branched chains with mixed lysine linkages (Rotin and Kumar, 2009). Polyubiquitin chains regulate multiple cellular functions including endocytosis of membrane proteins, protein degradation, DNA repair and act as scaffolds for signaling complexes (Popovic et al., 2014; Grimsey et al., 2015; Grimsey and Trejo, 2016).

### NEDD4 Family E3 Ubiquitin Ligases and the Regulation of GPCRs

Ubiquitination of proteins requires covalent ligation of a ubiquitin moiety to the substrate protein, mediated by E2 or E3 ubiquitin ligases. In humans, there are 30 E2 ubiquitin ligases and over 600 E3 ubiquitin ligases that can be divided into 3 broad families: ~95% of these are part of the really interesting new gene (RING) domain containing E3 ubiquitin ligases. Additionally, there are the two smaller families, the E6-AP carboxy terminus (HECT) domain containing E3 ligases and the ring-between-ring ligases. The RING E3s act as adaptor molecules to bridge the substrate and the E2 ligase to facilitate substrate ubiquitination. Whereas, the HECT domain ligases contain a conserved cysteine residue required for the formation of a thioester bond with the C-terminus of ubiquitin and catalyzing substrate ubiquitination (Scheffner and Kumar, 2014). Within the HECT family E3 ligases there are nine neural precursor cell expressed, developmentally downregulated-4 (NEDD4) HECT-containing E3 ligases (Table 1), characterized by a conserved domain architecture (Figure 1B). Each NEDD4 E3 ligase possesses two to four WW domains that regulate protein interactions, an N-terminal C2-domain, that binds Ca^+2^ and phospholipids to regulate ligase localization and the catalytic HECT domain (Buetow and Huang, 2016) (Figure 1B). The activity of NEDD4 family E3 ligases is regulated in part by interactions between the WW motifs and C2 domains, although the mechanism of these interactions is unclear for all family members (Rotin and Kumar, 2009). NEDD4 ligases are the most studied subgroup of E3 ligases due to their involvement in the regulation of intracellular ion concentrations (Staub et al., 1996; Harvey et al., 2001; Kamynina et al., 2001; Lu et al., 2008), cell proliferation and growth (Cao et al., 2008; Fouladkou et al., 2008), and in viral budding (Morita and Sundquist, 2004). In addition to the well-documented role of ubiquitination and NEDD4 ligases in the regulation of subcellular trafficking they can also regulate GPCR induced signaling (Abagyan and Totrov, 1994; Marchese and Benovic, 2001; Tanowitz and Von Zastrow, 2002; Marchese et al., 2003; Bhandari et al., 2007; Oo et al., 2007, 2011; Wolfe et al., 2007; Shenoy et al., 2008, 2009; Berthouze et al., 2009; Hislop et al., 2009; Mines et al., 2009; Berlin et al., 2010; Malik and Marchese, 2010; Chen et al., 2011; Groer et al., 2011; He et al., 2011; Henry et al., 2011, 2012; Dores et al., 2012, 2015; Grimsey et al., 2015, 2018; Jean-Charles et al., 2016) (Table 1). NEDD4 ligases can be directly recruited to GPCRs via their WW domains, as seen for AIP4 binding to phosphorylated chemokine receptor CXCR4 (Marchese et al., 2003) or indirectly via adaptor proteins as seen for β_2_-AR, where β-arrestin-2 binds to phosphorylated receptor and NEDD4.1 binds to the PPxY motif in β-arrestin-2 (Shenoy et al., 2008). However, how GPCRs regulate NEDD4 family E3 ligase recruitment remains largely unknown. Receptor ubiquitination in turn leads to association with other adaptor proteins that can facilitate either ubiquitin-dependent or ubiquitin-independent lysosomal degradation (Dores and Trejo, 2018). For example, endocytosis of the μ-opioid receptors is temporally controlled by Smurf2 dependent ubiquitination, this step is controlled by activated and phosphorylated receptors with β-arrestins and epsin1 forming a cargo-to-coat communication system and checkpoint for downstream pathways (Henry et al., 2012). Another example is the protease activated receptor 1 (PAR1) which is activated by Thrombin. In this case, receptor ubiquitination is redundant for GPCR internalization and degradation. Instead, the α-arrestin ARRDC3 (Alpha-arrestin domain containing protein 3) recruits WWP2 to ubiquitinate and activate ALIX (ALG-interacting protein X) and regulate lysosomal sorting of PAR1, this pathway has also been shown to be conserved for the purinergic receptor P2Y_1_ (Dores et al., 2012). Intriguingly, the ubiquitin-independent process of PAR1 endo-lysosomal trafficking enabled the discovery of an atypical use for GPCR ubiquitination. In this case, NEDD4-2 can ubiquitinate PAR1 or P2Y1 to induce the recruitment and stabilization of an adaptor protein complex to induce vascular proinflammatory signaling. However, as with many NEDD4 E3 ligase family members, it is still unclear how NEDD4-2 is recruited to the GPCR.

**Table 1:** NEDD4 family E3 ligases.

**Name**	**Full/alternate names**	**Regulation of GPCRs**	**References**
NEDD4-1 (NEDD4)	Neural precursor cell expressed developmentally down-regulated protein 4-1	Lysosomal degradation	Shenoy et al., 2008, 2009; Berthouze et al., 2009;
NEDD4-2 (NEDD4L)	Neural precursor cell expressed developmentally down-regulated protein 4-2	Promotes p38 activation	Wolfe et al., 2007; Chen et al., 2011; Dores et al., 2012, 2015; Grimsey et al., 2015
	Neural precursor cell expressed, developmentally down-regulated 4-Like, E3 ubiquitin protein ligase		
WWP1 (AIP5)	WW domain-containing E3 ubiquitin protein ligase 1 or Atrophin-1-Interacting Protein 5	Not yet known to regulate GPCRs	N.D.
WWP2 (AIP2)	WW domain-containing E3 ubiquitin protein ligase 2 or Atrophin-1-Interacting Protein 2	Proteasomal degradation	Oo et al., 2007, 2011; Dores et al., 2012, 2015
SMURF1	SMAD Specific E3 ubiquitin protein ligase 1	Not yet known to regulate GPCRs	N.D.
	HECT-Type E3 ubiquitin transferase SMURF1		
SMURF2	SMAD Specific E3 ubiquitin protein ligase 2	Clathrin-coated vesicle mobilization	Groer et al., 2011; He et al., 2011; Henry et al., 2011, 2012
	HECT-Type E3 ubiquitin transferase SMURF2		
NEDL1 (HECW1)	Nedd4-Like E3 Ubiquitin-protein ligase 1	Not yet known to regulate GPCRs	N.D.
	HECT, C2 And WW domain-containing protein 1		
NEDL2 (HECW2)	Nedd4-Like E3 ubiquitin-protein ligase 2	Not yet known to regulate GPCRs	N.D.
	HECT, C2 and WW domain-containing protein 2		
AIP4 (ITCH)	Atrophin-1-interacting protein 4 Or Itch E3 ubiquitin protein ligase	Lysosomal Degradation/Proteolytic Processing in MVBs	Abagyan and Totrov, 1994; Marchese and Benovic, 2001; Tanowitz and Von Zastrow, 2002; Marchese et al., 2003; Bhandari et al., 2007; Hislop et al., 2009; Mines et al., 2009; Berlin et al., 2010; Malik and Marchese, 2010; Henry et al., 2011, 2012

*A list of the nine NEDD4 family members and their respective roles in GPCR functions*.

### Ubiquitin Driven Atypical p38 Signaling in the Endocytic Pathway

Discovered in 2015, the ubiquitin-dependent stabilization and activation of the atypical mitogen activated protein kinase (MAPK) p38 signaling pathway by GPCRs represents a new use of GPCR ubiquitination, while providing fundamental insight into the regulation of p38 signaling (Grimsey et al., 2015) (Figure 1A). MAPK p38 is a ubiquitous target in pharmaceutical research, due to its critical role in regulating the biosynthesis and activation of many key proinflammatory cytokines in response to infection, cellular stress and more recently in the regulation of neurological disorders (Millan et al., 2011; Xing, 2015; Lee and Kim, 2017). However, despite extensive research, p38 inhibitors have been overwhelmingly unsuccessful in the clinic. This is in part attributed to the majority of the inhibitors targeting the highly conserved catalytic sites and ATP-binding pockets of the four p38 isoforms (p38α, β, δ, and γ), and the broad physiological and pathophysiological roles of p38α. Atypical p38 signaling represents a relatively unexplored branch of the p38 signaling pathway, and has recently been demonstrated to play a key role in the control of several clinically relevant diseases, such as ischemia reperfusion injury, myocardial ischemia, cardiotoxicity, and inflammation; including GPCR induced inflammation and vascular leakage (Tanno et al., 2003; Wang et al., 2013; Grimsey et al., 2015, 2018, 2019; Theivanthiran et al., 2015; De Nicola et al., 2018). Therefore, defining the mechanisms that mediate GPCR induced atypical p38 signaling could provide new insight into the development of novel p38 targeted therapeutics.

While it is well-known that many GPCRs can induce p38 signaling, in many cases it is not known exactly how they induce p38 activation. In the classical pathway p38 is activated by a three-tier kinase cascade, requiring the phospho-activation of the critical upstream kinase pair, MAPKK3 and MAPKK6 (MKK3/6), which in turn activate p38 via phosphorylation of the active loop (Raingeaud et al., 1996). Classical, MKK3/6 dependent p38 activation is commonly assumed to regulate all the identified p38 signaling responses. However, p38 can also be activated through two atypical pathways, independent from MKK3/6. In the first example, the Src family kinase, ZAP-70 binds and phosphorylates p38α and p38β, inducing a conformational change that enables p38 autophosphorylation, which is critical for T-cell activation (Salvador et al., 2005). In the second example, p38 recruits two adaptor proteins, TAB1 and TAB2 (Transforming growth factor beta, (TGFβ) Activated kinase 1 (TAK1) binding protein 1 and 2) (Ge et al., 2002) both of which are critical for the activation of TAK1 and the regulation of the NFκB inflammatory signaling pathway (Kanayama et al., 2004; Xia et al., 2009). The direct binding of TAB1 to p38α causes a conformational change in p38 that again enables p38 autophosphorylation of the active loop (De Nicola et al., 2013, 2018; Thapa et al., 2018). PAR1 and P2Y_1_ have been shown to be ubiquitinated by NEDD4-2, creating a K63-linked polyubiquitin chain that is essential for the recruitment of TAB2. TAB2 contains two ubiquitin binding motifs, a N-terminal conjugation to endoplasmic reticulum-associated degradation (CUE) ubiquitin binding domain, shown to specifically bind mono-ubiquitinated proteins (Shih et al., 2003; Kishida et al., 2005), and a C-terminal Npl4 zinc finger (NZF) ubiquitin binding domain, that specifically binds to at least two consecutive, K63-linked ubiquitin moieties (Kulathu et al., 2009). TAB2 recruitment and activation of the atypical signaling pathway can be blocked, by either the use of a ubiquitin deficient PAR1 receptor or mutation of the NZF domain of TAB2, demonstrated using biochemical co-association assays and live cell TIRF imaging showing a blockade of NZF mutant TAB2 recruitment to activated PAR1 (Grimsey et al., 2015). Interestingly, while PAR1 recruits wild-type TAB2 to small endocytic structures within 81 s of receptor activation, it is not known when TAB1 is recruited to the complex. Furthermore, blockade of receptor internalization, using either the dynamin ATPase inhibitor Dyngo-4a (McCluskey et al., 2013) or siRNA depletion of the endocytic clathrin adaptor proteins AP-2 and epsin-1, delayed PAR1 internalization and enhanced NEDD4-2 recruitment to the plasma membrane. However, blockade of PAR1 internalization did not inhibit p38 activation but instead prolonged p38 activity and enhanced receptor ubiquitination (Grimsey et al., 2018). Although not definitively proven, these data support a model where the stimulation of PAR1 rapidly induces the activation and recruitment of NEDD4-2, most likely at the plasma membrane. The ubiquitinated PAR1 mediates the endosomal recruitment of TAB2 and initiates the formation of a TAB1-p38α signaling complex. Before activation of the atypical pathway TAB1 undergoes rapid degradation at the proteasome (Grimsey et al., 2015; Theivanthiran et al., 2015). However, once recruited to the signaling complex, p38 phosphorylates and stabilizes TAB1 expression and recent studies have shown that phosphorylated TAB1 then remains bound to the active p38 (Grimsey et al., 2015; De Nicola et al., 2018). Thus, p38-phosphorylated TAB1 potentially provides a mechanism for cells to differentially regulate transcriptional activation by the MKK3/6 and TAB1 dependent pathways through spatial restriction of p38 translocation to the nucleus and sequestration in the cytosol (Lu et al., 2006; Grimsey et al., 2015). Raising the question of how GPCRs initiate activation of NEDD4 family E3 ligases, specifically; how does PAR1 regulate activation of NEDD4-2?

### GPCR Mediated Activation of NEDD4-2 and Endosomal Signaling

An emerging concept in understanding how the catalytic activity of the NEDD4 family E3 ligases are regulated is that intramolecular interactions can hold the E3 ligases in inactive/inhibited confirmations that can only be released by phosphorylation or accessory protein binding. An example of this is where serine phosphorylation of NEDD4-2 (Debonneville et al., 2001; Bhalla et al., 2005; Ichimura et al., 2005), regulates the recruitment of the inhibitory protein 14-3-3, blocking NEDD4-2 interaction with the ENaC (epithelial sodium channel) preventing its ubiquitination and subsequent degradation (Figure 1C). However, the activating phosphatase that dephosphorylates NEDD4-2 has not been identified. Additionally, NEDD4-2 activity is thought to be restricted by the WW domains binding inter- or intramolecularly to a PPxY motif in the HECT domain, restricting auto-ubiquitination and degradation (Bruce et al., 2008). However, a recent structural study showed that Ca^2+^ binding was essential for the active confirmation of NEDD4-2 and that the interaction between WW3 and the HECT domain requires partial unfolding of the HECT domain; suggesting that this mechanism regulates degradation of old or damaged E3 ligases (Escobedo et al., 2014). Therefore, it is unlikely to regulate GPCR induced activation of NEDD4-2. Instead, endothelial NEDD4-2 is activated by a tyrosine switch in an auto-inhibitory peptide (Grimsey et al., 2018). In this case, activation of PAR1 or P2Y_1_ triggers G-protein dependent (predominantly through Gα_q_) mediated activation of c-Src (Figure 1A). Activated c-Src is then able to phosphorylate NEDD4-2 at a critical tyrosine residue (Y485), situated on a linker peptide between WW domain 2 and 3 (Grimsey et al., 2018). Importantly, this linker peptide (2,3-linker peptide) has previously been shown to be critical for regulating the activation of other NEDD4 family E3 ligases, including AIP4, WWP1, WWP2, and NEDD4.1 (Chen et al., 2017; Zhu et al., 2017), but not NEDD4-2. Zhu et al. demonstrated that WW2 and the 2,3-linker peptide of AIP4 locks the ligase in an auto-inhibited state, through binding to the catalytic HECT domain. The HECT domain has a bilobal domain structure consisting of a C-lobe and N-lobe connected by a flexible hinge loop. The 2, 3-linker peptide wraps around the N-lobe and “locks” the C-lobe in the closed confirmation, by restricting movement of the flexible hinge loop (Zhu et al., 2017). Release of this inhibitory state was found to be regulated by binding of the NEDD4 family of interacting protein 1 (Ndfip1) or JNK1 mediated phosphorylation of the C-terminal proline rich region, both of which have been shown to occur in activated T-cells (Gao et al., 2004; Gallagher et al., 2006; Oberst et al., 2007). In a separate study, Chen et al. demonstrated that in addition to AIP4, WWP1, WWP2, and NEDD4-1 all contained a conserved alpha helical 2,3-linker peptide that uses the same method as described above to lock their respective HECT domains in an auto-inhibited state. Furthermore, they show that tyrosine phosphorylation of the linker peptide could release its binding to the HECT domain and induce full ligase activation (Chen et al., 2017), however no tyrosine kinase was identified. Structural modeling of NEDD4-2, suggests that Y485 interacts with an acidic triad (891-DAE-893) in the C-lobe similar to the interactions seen for AIP4 and WWP2 (Grimsey et al., 2018). It is therefore predicted that the 2,3-linker peptide of NEDD4-2 holds the ligase in a closed auto-inhibited confirmation. In this case NEDD4-2 auto-inhibition is released by GPCR induced activation of c-Src and tyrosine phosphorylation of Y485, disrupting the interaction through electrostatic repulsion and enabling the “open” active confirmation of NEDD4-2. Phosphorylation of NEDD4-2 Y485 by c-Src was further shown to be critical for both thrombin and ADP induced activation of endosomal p38 signaling and induction of PAR1 induced endothelial barrier disruption (Grimsey et al., 2018). While it was shown that c-Src is rapidly activated at the plasma membrane, and that blockade of receptor internalization did not stop NEDD4-2 recruitment and activation, it is still unclear whether NEDD4-2 activation occurs solely at the plasma membrane or whether endocytosed GPCRs can recruit NEDD4-2 to the early endosome. Additionally, it is also important to investigate whether c-Src can regulate the activation of the other NEDD4 family E3 ligase that are critical for GPCR trafficking and degradation, including AIP4, NEDD4.1 and WWP1 (Ellis et al., 1999; Marchese et al., 2003; Dores et al., 2015; Alekhina and Marchese, 2016).

### Physiological Importance of Endosomal MAPK p38 Signaling

Thrombin and ADP rapidly induce the activation of ubiquitin-dependent proinflammatory p38 signaling by activation of PAR1 and P2Y_1_. Atypical p38 signaling has been shown to be critical for the induction of endothelial barrier permeability *in vitro* and vascular leakage *in vivo*, and is likely a major contributor to GPCR dependent tissue edema through regulation of endothelial cell adherens junctions and actin cytoskeleton remodeling (Grimsey et al., 2015, 2018). However, this process is still poorly understood and further investigation is required to determine the specific mechanisms that regulate atypical p38 signaling induced barrier disfunction. Intracerebral hemorrhage (ICH) induces the activation by thrombin, which leads to life-threatening cerebral edema (Liu et al., 2010). Interestingly, rapid administration of a Src antagonist following ICH blocks edema, whereas delayed administration prevented repair of the blood brain barrier (BBB). It is unknown whether Src dependent p38 signaling could be a contributing factor in BBB dysfunction and whether targeting p38 signaling may be a viable therapeutic target in the treatment of ICH and BBB repair.

The discovery of the ubiquitin driven endosomal signaling pathway was facilitated by the identification of the ubiquitin-independent lysosomal sorting of PAR1 (Dores et al., 2012), interestingly multiple other GPCRs contain this conserved motif (Dores et al., 2012). It is tempting to speculate that this ubiquitin-independent endo-lysosomal sorting pathway exists in part to enable the activation of ubiquitin-dependent atypical p38 signaling. It will be critical for future research to identify which GPCRs utilize this pathway and which proinflammatory signaling responses are activated.

Targeting of the atypical p38 signaling pathway represents a novel, presently understudied therapeutic approach for the regulation of proinflammatory p38 signaling. The interaction between p38 and TAB1 is specifically required for the amplification of atypical p38 signaling (De Nicola et al., 2013, 2018; Wang et al., 2013; Grimsey et al., 2015; Pei et al., 2018; Thapa et al., 2018). Genetic mutation of the 4 critical residues on TAB1 [TAB1 knock-in (KI)] required for this interaction protect mice from myocardial ischemic injury. Unlike the p38 and TAB1 knock out models, which are both embryonic lethal (Adams et al., 2000; Komatsu et al., 2002), TAB1-KI mutations display no developmental defects, suggesting that targeting the TAB1-p38 interface will not have the same complications seen for the current p38 inhibitors. Moreover, the structural analysis of the p38-TAB1 interface revealed a hydrophobic pocket in p38, which can be targeted by functionalized 3-amino-1-adamantanol. Critically, binding of 3-amino-1-adamantanol blocked p38 kinase activity in an *in vitro* kinase assay (De Nicola et al., 2018). Thus, demonstrating that targeting the TAB1-p38 interface with small molecule inhibitors could be a viable therapeutic approach to bypass the current failures seen for ATP-competitive p38 inhibitors.

In summary, it is clear that GPCRs can induce a proinflammatory p38 signaling pathway as they undergo endocytic trafficking, with induction of this pathway being mediated by GPCR induced c-Src dependent tyrosine phosphorylation of the E3 ligase NEDD4-2. While significant steps have been made into defining the molecular regulation of this pathway, further investigations are critical and are likely to reveal novel therapeutic targets. Furthermore, peptide inhibitors or small molecules targeting the TAB1-p38 interaction offer a potentially paradigm shifting approach to the treatment of chronic proinflammatory diseases.

## Author Contributions

JB and NG wrote and edited the manuscript. NG created the figure.

### Conflict of Interest Statement

The authors declare that the research was conducted in the absence of any commercial or financial relationships that could be construed as a potential conflict of interest.
